# 

**Published:** 1866-08

**Authors:** 


### CONTENTS.

ORIGINAL COMMUNICATIONS.

Filling Teeth,........ 331
Professional Character........ 333
Alveolar Abcess........ 340
Code of Dental Ethics........ 343

PROCEEDINGS OF SOCIETIES.

Indiana State Dental Association........ 346

SELECTIONS.

A Description of Professor Vander Weyde's Apparatus, for the Generation and Administration of Nitrous-Oxide and other Anæsthetic Gases........ 358
Local Anæsthesia........ 362
On Anæsthetics........ 363
Removal of Upper Maxillary Bone for Myeloid disease. Palliative operation two years before........ 366
Use and Abuse of Poultices........ 369
Removal of the entire tongue successfully during life........ 371
Effects of Tobacco on the Mental Powers........ 371
Ether........ 371

EDITORIAL.

Organization of the Ohio State Dental Association........ 372
A Prize........ 373
Ho! Every one to the Breeches!!........ 374
Obituary—Dr. Slack........ 376

